# Supplementary material for: Stress responsive miR-31 is a major modulator of mouse intestinal stem cells during regeneration and tumorigenesis
Source: eLife. 2017 Sep 5;6:e29538. doi: 10.7554/eLife.29538 (PMC5584991; doi:10.7554/eLife.29538)
Supplement: Transparent reporting form [file elife-29538-transrepform.docx]

***eLife’s* transparent reporting form**

We encourage authors to provide detailed information *within their submission* to facilitate the interpretation and replication of experiments. If you have any questions, please contact us: [editorial@elifesciences.org](mailto:editorial@elifesciences.org).

**Sample-size estimation**

- You should state whether an appropriate sample size was computed when the study was being designed
- You should state the statistical method of sample size computation and any required assumptions
- If no explicit power analysis was used, you should describe how you decided what sample (replicate) size (number) to use

Please outline where this information can be found within the submission (e.g., page numbers or figure legends), or explain why this information doesn’t apply to your submission:

All analyses were performed in triplicate or greater and the means obtained were used for independent t-tests. All data were obtained from 8-weeks mice expect special instructions and all date were reported as averaged normalized intensity with SD(standard deviation).

Most sample size were found within figure legends.

For the relative miR-31 expression in Figure 1A, n=4 mice in each group.

For the miR-31 in situ in Figure 1B, n=3 mice in each group.

For the relative miR-31 expression in Figure 1C, n=3 mice in each group.

For the miR-31 in situ in Figure 1D, n=3 mice in each group.

For the body weight and intestine length in Figure 1E, n=6 mice in each group.

For the crypt height in Figure 1F, n=3 biological replicates and each mouse count 20 crypts.

For the number of Ki67+ cells/crypt in Figure 1G, n=3 biological replicates and each mouse count 20 crypts.

For the number of Casp3+ cells/villus in Figure 1H, n=3 biological replicates and each mouse count 60 villi.

For the crypt height in Figure 1I, n=3 biological replicates and each mouse count 20 crypts.

For the percentage of GFP+ cells in Figure 2A, 2B and 2C, n=4 biological replicates.

For the percentage of EdU+/GFP+ cells in Figure 2D, n=3 biological replicates.

For the date in Figure 2E, 6 weeks mice were used in 5 biological replicates, and each mouse had 3 technical replicates.500 crypt were seaded in each pool.

For the Lgr5+ cells lineage tracing in Figure 2F and 2G, n=3 biological replicates and each mouse count 20 crypts or views.

For the *Hopx* level in Figure 2H, n=3 biological replicates.

For the Hopx+ cells lineage tracing in Figure 2I, n=3 biological replicates.

For the percentage of Casp3+ at indicated positions in Figure 2J, n=3 biological replicates and each mouse count 50 crypts.

For the date in Figure 2K, 6 weeks mice were used in 3 biological replicates, 500 crypt were seaded in each pool. 10 organoids were counted in each pool.

For the Ki67+crypt or Ki67+ cells per crypt in Figure 3A, n=5 independent experimental repeats and each mouse count 20 crypts or views.

For the number of Casp3+ cell/crypt or the percentage of Casp3+ at indicated positions in Figure 3B, n=3 biological replicates and each mouse count 50 crypts.

For the *Lgr5* level in Figure 3C, n=3 biological replicates.

For the Lgr5+ cells lineage tracing in Figure 3D, n=3 biological replicates.

For the date in Figure 3E, n=3 biological replicates and each mouse count 20 crypts or views.

For the Hopx+ cells lineage tracing in Figure 3F, n=3 biological replicates.

For the LacZ+ crypts per 1400μm in Figure 3G, n=3 independent experimental repeats and each mouse count 20 fields.

For the LacZ+/Ki67+ cells per crypt in Figure 3H, n=3 independent experimental repeats and each mouse count 20 crypts.

For the LacZ+ cells per crypt in Figure 4A and 4B, n=3 biological replicates and each mouse count 20 crypts.

For the relative expression level in Figure 4C, n= 3 biological replicates.

For the protein expression level in Figure 4D, n= 3 biological replicates.

For the relative expression level in Figure 4E, n= 3 biological replicates.

For the protein expression level in Figure 4F, n= 3 biological replicates.

For the relative expression level in Figure 4G, n= 4 biological replicates.

For the relative expression level in Figure 4H, n= 3 biological replicates.

For the luciferase activity in Figure 4I, n= 3 biological replicates.

For the date in Figure 4J and 4K, n= 3 biological replicates.

For the date in Figure 4L, n= 4 biological replicates.

For the protein expression level in Figure 5A, n= 3 biological replicates.

For the relative expression level in Figure 5B-5E, n= 3 biological replicates.

For the luciferase activity in Figure 5F and 5G, n= 3 biological replicates.

For the organoid forming efficiency in Figure 5H and 5I, n= 3 biological replicates.

For the relative expression level in Figure 6A-6C, n= 3 biological replicates.

For the protein expression level in Figure 6D and 6E, n= 3 biological replicates.

For the relative expression level in Figure 6F and 6G, n= 3 biological replicates.

For the relative expression level in Figure 6H-6J, n= 3 biological replicates.

For the tumor volume and weight in Figure 7A, there were five tumors in mimics-NC, miR-31 mimics and miR-31 inhibitor group, while 4 tumors in inhibitor-NC group.

For the ki67+ and cyclinD1+ cells in Figure 7B, each tumor were counted 3 fields respectively.

For tumor number and volume in Figure 7C, n=6 mice per group. When tumor number in a mouse >10, we count 10 tumors volume at random; tumor number in a mouse <10, we count all.

For the date in Figure 7D-7F, n= 3 biological replicates.

For tumor number and volume in Figure 7G, n=6 mice per group.

For the date in Figure 7H-7J, n= 6 biological replicates.

For the relative expression level in Figure 8B, n= 3 biological replicates.

For the protein expression level in Figure 8C, n= 3 biological replicates.

For the immunohistochemistry in Figure 8D, n= 3 biological replicates.

For the relative expression level in Figure 8E, n= 3 biological replicates.

For the protein expression level in Figure 8F, n= 3 biological replicates.

For the miR-31 expression level in Figure 8G, n= 3 biological replicates.

For the protein expression level in Figure 8H, n= 3 biological replicates.

For the miR-31 expression level in Figure 8I, n= 3 biological replicates

For the protein expression level in Figure 8J, n= 3 biological replicates.

For the miR-31 expression level in Figure 8K, n= 3 biological replicates

For the relative luciferase activity in Figure 8L, n= 3 biological replicates

For the date in Figure 8M, n= 3 biological replicates

For the miR-31 expression level in Figure 1-figure supplement 1B,1E and 1G, n=3 independent experimental repeats.

For the miR-31 in situ in Figure 1-figure supplement 1H, n=3 mice in each group.

For the length of villi or crypt/villius in Figure 1-figure supplement 2A, n= 3 biological replicates.

For the crypt height in Figure 1-figure supplement 2B, n= 3 biological replicates.

For the crypt height in Figure 1-figure supplement 2C-E, n= 3 biological replicates respectively.

For the immunohistochemistry of Ki67 and Casp3 in Figure 1-figure supplement 3A-B, n= 3 biological replicates.

For the immunohistochemistry of GFPand the number of GFP+ cells per crypt in Figure 1-figure supplement 3C, n= 3 biological replicates.

For the percentage of GFP+ cells in Figure 1-figure supplement 3D, n= 3 biological replicates.

For the date in Figure 1-figure supplement 4A and 4B, n= 3 biological replicates.

For the body weight and intestine length in Figure 1-figure supplement 5A, n=4 mice in each group.

For the date in Figure 1-figure supplement 5B, n=266 mice.

For the date in Figure 1-figure supplement 5C-D, n= 3 biological replicates.

For the date in Figure 1-figure supplement 6A and B, n= 3 biological replicates.

For the crypt height in Figure 1-figure supplement 7A, n= 4 biological replicates.

For the immunohistochemistry of Ki67 and the number of Ki67+ cells per crypt in Figure 1-figure supplement 7B, n= 3 biological replicates.

For the immunohistochemistry of Casp3 and the number of Casp3+ cells in Figure 1-figure supplement 7C and 7D, n= 3 biological replicates and each mouse count 20 crypts.

For the immunohistochemistry of BrdU in Figure 1-figure supplement 8, n= 3 biological replicates at each points.

For the GFP+ cells per crypt in Figure 2-figure supplement 1A, n=4 independent experimental repeats and each mouse count 50 crypts.

For the date in Figure 2-figure supplement 1B, 6 weeks mice were used in 5 independent experiment, and 10 organoids were counted in each mouse.

For the Lgr5+ cells lineage tracing in Figure 2-figure supplement 1C, n=3 biological replicates and each mouse count 20 views.

For the date in Figure 2-figure supplement 1D and 1E, n=3 biological replicates and each mouse count 20 crypt or views.

For the date in Figure 2-figure supplement 1F, n=3 biological replicates.

For the immunohistochemistry of Casp3 in Figure 2-figure supplement 1G, n=3 biological replicates.

For the date in Figure 3-figure supplement 1A, n=3 biological replicates.

For the date in Figure 3-figure supplement 1B, n=4 biological replicates and each mouse count 20 crypt or views.

For the date in Figure 3-figure supplement 1C, n=3 biological replicates.

For the date in Figure 3-figure supplement 1D, n=3 biological replicates.

For the date in Figure 3-figure supplement 1E, n=3 biological replicates and each mouse count 20 crypt or views.

For the immunohistochemistry of β-Catenin and the number of nuclear β-Catenin + cells per crypt in Figure 4-figure supplement 1A and 1B, n= 3 biological replicates and each mouse count 20 crypts.

For the protein expression level in Figure 4-figure supplement 1C, n= 3 biological replicates.

For the date in Figure 5-figure supplement 1A and 1B, n=3 biological replicates.

For the protein expression level in Figure 5-figure supplement 1C and 1D, n= 3 biological replicates.

For the date in Figure 6-figure supplement 2A -2C, n=3 biological replicates.

For the protein expression level in Figure 6-figure supplement 3A and 3B, n= 3 biological replicates.

For the MTT proliferation in Figure 7-figure supplement 1A, n= 3 technical replicates.

For the date in Figure 7-figure supplement 1B, n=3 biological replicates.

For the date in Figure 7-figure supplement 1C, n= 3 biological replicates and each mouse count 20 crypts.

For the date in Figure 7-figure supplement 1D, n= 3 biological replicates.

**Replicates**

- You should report how often each experiment was performed
- You should include a definition of biological versus technical replication
- The data obtained should be provided and sufficient information should be provided to indicate the number of independent biological and/or technical replicates
- If you encountered any outliers, you should describe how these were handled
- Criteria for exclusion/inclusion of data should be clearly stated
- High-throughput sequence data should be uploaded before submission, with a private link for reviewers provided (these are available from both GEO and ArrayExpress)

Please outline where this information can be found within the submission (e.g., page numbers or figure legends), or explain why this information doesn’t apply to your submission:

All experiment were performd three or more times except special instruction.

For biological replication, sample mice were prepared on the same age and sample cells were prepared on the different days.

For technical replication,multiple tissue sections of one mouse with differern antibody were stained under the same condition.

Before every experiment, the laser power was measured first adjusted to the fixed value for consistent imaging conditions.

For Figure 1A, the experiment were perform 4 times, n=4 biological replication and 3 technical replicates in each tissue ..

For Figure 1B, the experiment were perform 3 times, n=3 biological replication.

For Figure 1C, the experiment were perform 3 times, n=3 biological and 3 technical replicates in each tissue.

For Figure 1D, the experiment were perform 3 times, n=3 biological replication

For Figure 1E, the experiment were perform 6 times, n=6 biological replication.

For Figure 1F, the experiment were perform 3 times, n=3 biological replication.

For Figure 1G, the experiment were perform 3times, n=3 biological replication.

For Figure 1H, the experiment were perform 3 times, n=3 biological replication.

For Figure 1I, the experiment were perform 3 times, n=3 biological replication.

For Figure 2A-C, the experiment were perform 4 times, n=4 biological replication.

For Figure 2D, the experiment were perform 3 times, n=3 biological replication.

For Figure 2E, the experiment were perform 5 times, n=5 biological replication and 3 technical replicates in each mice.

For Figure 2F-G, the experiment were perform 3 times, n=3 biological replication.

For Figure 2H, the experiment were perform 3 times, n=3 biological replication and 3 technical replicates in each mice.

For Figure 2I, the experiment were perform 3 times, n=3 biological replication.

For Figure 2J, the experiment were perform 3 times, n=3 biological replication.

For Figure 2K, the experiment were perform 3 times, n=3 biological replication and 3 technical replicates in each mice.

For Figure 3A, the experiment were perform 5 times, n=5 biological replication.

For Figure 3B, the experiment were perform 3 times, n=3 biological replication.

For Figure 3C, the experiment were perform 3times, n=3 biological replication and 3 technical replicates in each mice..

For Figure 3D-3E, the experiment were perform 3 times, n=3 biological replication.

For Figure 3F, the experiment were perform 3 times, n=3 biological replication.

For Figure 3G-3H, the experiment were perform 3 times, n=3 biological replication and 3 technical replicates in each mice.

For Figure 4A-B, the experiment were perform 3 times, n=3 biological replication and 3 technical replicates in each tissue.

For Figure 4C-D, the experiment were perform 3 times, n=3 biological replication.

For Figure 4E-F, the experiment were perform 3 times, n=3 biological replication.

For Figure 4G, the experiment were perform 4times, n=4 biological replication.

For Figure 4H, the experiment were perform 3 times, n=3 biological replication.

For Figure 4I, the experiment were perform 3 times, n=3 biological replication.

For Figure 4J-4K, the experiment were perform 3 times, n=3 biological replication and 3 technical replicates in each mouse.

For Figure 4L, the experiment were perform 3 times, n=4 biological replication and 3 technical replicates in each mouse.

For Figure 5A, the experiment were perform 3 times, n=3 biological replication and 3 technical replicates in each tissue.

For Figure 5B-G, the experiment were perform 3 times, n=3 biological replication.

For Figure 5H-I, the experiment were perform 3 times, n=3 biological replication.

For Figure 6A-C, the experiment were perform 3 times, n=3 biological replication and 3 technical replicates in each tissue.

For Figure 6D-E, the experiment were perform 3 times, n=3 biological replication and 3 technical replicates in each tissue.

For Figure 6F, the experiment were perform 3 times, n=3 technical replicates.

For Figure 6G, the experiment were perform 3 times, n=3 technical replicates.

For Figure 6H, the experiment were perform 3 times, n=3 biological replication and 3 technical replicates in each tissue.

For Figure 6I-J, the experiment were perform 3 times, n=3 biological replication and 3 technical replicates in each tissue.

For Figure 7A, the inhibitor-NC grouo were perform 2 times, n=4 biological replication and 2 technical replicates in each time.The anti-miR31 group were perform 2 times, n=5 biological replication and 5 technical replicates in all.The mimics-NC and mimics group were perform 2 times, n=5 biological replication and 5 technical replicates in all.

For Figure 7B, the experiment were perform 3 times, n=3 biological replication.

For Figure 7C, the experiment were perform 2 times, n=6 biological replication and 3 technical replicates in each time.

For Figure 7D-7F, the experiment were perform 3 times, n=3 biological replication.

For Figure 7G-7H, the experiment were perform 2 times, n=6 biological replication.

For Figure 7I-7J, the experiment were perform 3 times, n=3 biological replication.

For Figure 8B, the experiment were perform 3 times, n=3 biological replication.

For Figure 8C-E, the experiment were perform 3 times, n=3 biological replication and 3 technical replicates in each tissue.

For Figure 8F-H, the experiment were perform 3 times, n=3 biological replication.

For Figure 8I, the experiment were perform 3 times, n=3 biological replication and 3 technical replicates in each tissue.

For Figure 8J, the experiment were perform 3 times, n=3 biological replication.

For Figure 8K, the experiment were perform 3 times, n=3 biological replication and 3 technical replicates in each tissue.

For Figure 8L-M, the experiment were perform 3 times, n=3 technical replicates.

For Figure1-figure supplement 1B,1E and 1G the experiment were perform 3 times, n=3 biological replication and 3 technical replicates in each tissue.

For Figure1-figure supplement 1H, the experiment were perform 3 times, n=3 biological replication.

For Figure1-figure supplement 2A, the experiment were perform 3 times, n=3 biological replication.

For Figure1-figure supplement 2B-2E, the experiment were perform 3 times, n=3 biological replication.

For Figure 1-figure supplement 3A-B, n= 3 biological replicates.

For Figure 1-figure supplement 3C-3D, n= 3 biological replicates and 3 technical replicates in each tissue.

For Figure supplement 4A and 4B, n= 3 biological replicates.

For Figure 1-figure supplement 5A, the experiment were perform 3 times, n=4 mice in each group.

For Figure 1-figure supplement 5B, n=266 mice in all.

For Figure 1-figure supplement 5C-D, n= 3 biological replicates.

For Figure 1-figure supplement 6A-B, n= 3 biological replicates.

For Figure 1-figure supplement 7A, n= 4 biological replicates.

For Figure 1-figure supplement 7B-D, n= 3 biological replicates.

For Figure 1-figure supplement 8, the experiment were perform 3 times, n= 3 biological replicates at each points.

For Figure 2-figure supplement 1A, the experiment were perform 4 times, n=4 mice and count 50 crypts.

For Figure 2-figure supplement 1B, 6 weeks mice were used in 5 independent experiment.

For Figure 2-figure supplement 1C, n=3 biological replicates.

For Figure 2-figure supplement 1D-E, n=3 biological replicates and each mouse count 20 crypt or views.

For Figure 2-figure supplement 1F, n=3 biological replicates.

For Figure 2-figure supplement 1G, n=3 biological replicates.

For Figure 3-figure supplement 1A, n=3 biological replicates.

For Figure 3-figure supplement 1B, the experiment were perform 3 times, n=4 biological replicates and each mouse count 20 crypt or views.

For Figure 3-figure supplement 1C, the experiment were perform 3 times, n=3 biological replicates.

For in Figure 3-figure supplement 1D, the experiment were perform 3 times ,n=3 biological replicates.

For Figure 3-figure supplement 1E, n=3 biological replicates and each mouse count 20 crypt or views.

For Figure 4-figure supplement 1A-1B, n= 3 biological replicates and each mouse count 20 crypts.

For Figure 4-figure supplement 1C, the experiment were perform 3 times, n= 3 biological replicates.

For Figure 5-figure supplement 1A-1B, n=3 biological replicates.

For Figure 5-figure supplement 1C-D, the experiment were perform 3 times, n= 3 biological replicates.

For Figure 6-figure supplement 2A-C, n=3 biological replicates.

For Figure 6-figure supplement 3-B, the experiment were perform 3 times, n= 3 biological replicates.

For Figure 7-figure supplement 1A, n= 3 technical replicates.

For Figure 7-figure supplement 1B, n=3 biological replicates.

For Figure 7-figure supplement 1C, n= 3 biological replicates and each mouse count 20 crypts.

For Figure 7-figure supplement 1D, n= 3 biological replicates.

**Statistical reporting**

- Statistical analysis methods should be described and justified
- Raw data should be presented in figures whenever informative to do so (typically when N per group is less than 10)
- For each experiment, you should identify the statistical tests used, exact values of N, definitions of center, methods of multiple test correction, and dispersion and precision measures (e.g., mean, median, SD, SEM, confidence intervals; and, for the major substantive results, a measure of effect size (e.g., Pearson's r, Cohen's d)
- Report exact p-values wherever possible alongside the summary statistics and 95% confidence intervals. These should be reported for all key questions and not only when the p-value is less than 0.05.

All analyses were performed in triplicate or greater and the means obtained were used for independent t-tests. Asterisks denote statistical significance (*P < 0.05; **P < 0.01; ***P<0.001). All data are reported as mean ± SD. Means and standard errors from at least three independent experiments are presented in all graphs.

Please outline where this information can be found within the submission (e.g., page numbers or figure legends), or explain why this information doesn’t apply to your submission:

(For large datasets, or papers with a very large number of statistical tests, you may upload a single table file with tests, Ns, etc., with reference to page numbers in the manuscript.)

**Additional data files (“source data”)**

- We encourage you to upload relevant additional data files, such as numerical data that are represented as a graph in a figure, or as a summary table
- Where provided, these should be in the most useful format, and they can be uploaded as “Source data” files linked to a main figure or table
- Include model definition files including the full list of parameters used
- Include code used for data analysis (e.g., R, MatLab)
- Avoid stating that data files are “available upon request”

Please indicate the figures or tables for which source data files have been provided:

Data and statistic values for all graphs in main figures are provided with an Excel worksheet file (source data file).
